# Pressure-Induced Metallization of BaH_2_ and the Effect of Hydrogenation

**DOI:** 10.1021/acs.jpclett.3c02704

**Published:** 2023-12-12

**Authors:** Hannah A. Shuttleworth, Israel Osmond, Calum Strain, Jack Binns, Jonathan Buhot, Sven Friedemann, Ross T. Howie, Eugene Gregoryanz, Miriam Peña-Alvarez

**Affiliations:** † Centre for Science at Extreme Conditions, The University of Edinburgh, Edinburgh EH8 8AQ, United Kingdom; ‡ Center for High Pressure Science and Technology Advanced Research, 1690 Cailun Road, Shanghai 201203, People’s Republic of China; § H.H. Wills Physics Laboratory, University of Bristol, Bristol BS8 1TL, United Kingdom; ∥ Key Laboratory of Materials Physics, Institute of Solid State Physics, Chinese Academy of Sciences (CAS), Hefei, Anhui 230031, People’s Republic of China

## Abstract

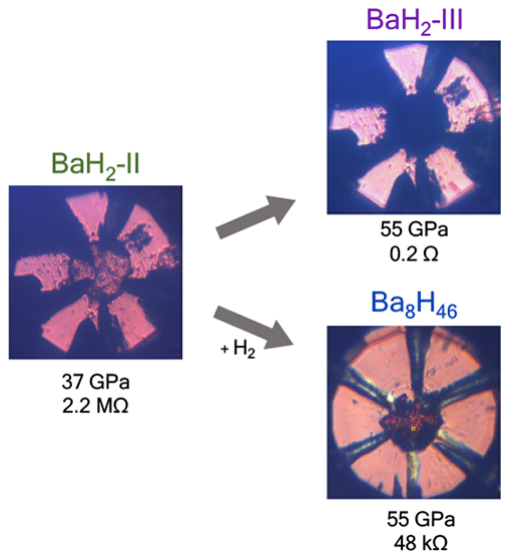

Using optical spectroscopy, X-ray diffraction, and electrical transport measurements, we have studied the pressure-induced metallization in BaH_2_ and Ba_8_H_46_. Our combined measurements suggest a structural phase transition from BaH_2_-II to BaH_2_-III accompanied by band gap closure and transformation to a metallic state at 57 GPa. The metallization is confirmed by resistance measurements as a function of the pressure and temperature. We also confirm that, with further hydrogenation, BaH_2_ forms the previously observed Weaire–Phelan Ba_8_H_46_, synthesized at 45 GPa and 1200 K. In this compound, metallization pressure is shifted to 85 GPa. Through a comparison of the properties of these two compounds, a question is raised about the importance of the hydrogen content in the electronic properties of hydride systems.

High pressure can be effectively used to convert insulating materials to metals^1−4^ and metals to insulators,^5,6^ producing compounds that are otherwise unstable under ambient conditions. In recent years, the use of high pressure to synthesize novel hydrogen-rich compounds has grown exponentially, facilitating the synthesis of unreported electronic states.^3,7−14^ Specifically, the recent discoveries of superconducting hydrides having critical temperatures approaching room temperature have led to a renewed interest in binary hydrides.^7−9,13,15−18^ The hydrogen-assisted superconductivity under pressure is referred to as “chemical pre-compression” of molecular hydrogen, where the light mass of hydrogen provides high-frequency phonons, enhancing the coupling to electrons.^19^ Although it has been proven that hydrogen can, in fact, be beneficial for electron–phonon coupling,^7,8,10,11,13^ the relationship between the hydrogen content and pressure required to reach such metallic superconducting states is not always obvious.

The alkaline earth elements form dihydrides (MH_2_, with M being the metal), which are stable at ambient conditions and are composed of the ions M^+2^ and 2H^–^.^20^ The heavy alkaline earth dihydrides, namely, CaH_2_, SrH_2_, and BaH_2_, are predicted to become metallic following a series of structural transitions.^20−24^ The metallization pressure of such compounds is expected to decrease with an increasing metal radius, which correlates with increasing electronegativity.^20,25^ Specifically, the predicted metallization pressure is 102 GPa (*T*_c_ = 1 mK) for CaH_2_, 177 GPa for SrH_2_, and 50 GPa for BaH_2_ (*T*_c_ = 13 mK).^20−24,26^ Neither the metallic nor superconducting transitions of these alkaline earth dihydrides have been experimentally observed.

The alkaline earth polyhydrides have also been suggested as candidates for high *T*_c_ superconductivity. For example, CaH_6_ was predicted to exhibit a superconducting critical temperature of 220–235 K at 150 GPa.^17^ This was later experimentally confirmed, with a superconducting transition observed to occur at 210 K and 160–190 GPa.^15,16^ In the Sr–H system, a range of strontium polyhydrides have been synthesized above 70 GPa: SrH_9_, SrH_6_, and SrH_22_, of which the latter two are expected to metallize at 220 and 200 GPa, respectively.^12^ SrH_22_ is predicted to be superconducting at 200 GPa and 21 K, and Sr_3_H_13_ is predicted to exhibit superconductivity at 150 GPa and 84 K.^12^ Numerous barium polyhydride polymorphs have also been discovered, with Ba_8_H_46_ stable at 50 GPa,^27^ while BaH_4_, BaH_6_, BaH_10_, and BaH_12_ are all stable above 90 GPa.^11,25,28^ Most notably, BaH_12_ has been demonstrated to exhibit superconductivity at 20 K and 140 GPa.^11^ However, the electrical transport properties of the other barium hydrides have not yet been measured experimentally. Hence, there is limited information about how hydrogen incorporation affects the pressure of metallization.

BaH_2_, with the lowest predicted pressure of metallization of the alkaline earth hydrides, is an ideal candidate to study the effects of hydrogenation. Previous high-pressure studies of BaH_2_ report two structural phase transitions upon compression: from an insulating, cotunnite (*Pnma*) structure (BaH_2_-I) to a hexagonal close-packed Ni_2_In-type (*P*6_3_/*mmc*) structure at 1.6 GPa (BaH_2_-II)^21,23,29^ and at 54 GPa from BaH_2_-II to an AlB_2_-type simple hexagonal (SH) structure (BaH_2_-III). Ionic (H^–^) transport measurements have been measured previously up to 11 GPa, in which strikingly high ionic conductivity was measured.^30^ Additionally, it has been predicted that BaH_2_-III is metallic, but this has not been experimentally realized.^21,22^ Furthermore, when BaH_2_ is compressed in a H_2_ medium, it incorporates H_2_ molecules, forming BaH_4_ by 40 GPa.^25,27^ Here, the bonding nature of H_2_ is altered by the pre-compression provided by Ba^2+^, inducing the elongation of the H_2_ bond. Not only that, but BaH_2_ undergoes further hydrogenation into a Weaire–Phelan barium polyhydride (Ba_8_H_46_) and forms a clathrate by 50 GPa, which stabilizes tetrahedral cages that fit only one hydrogen atom, thus dissociating the H_2_ molecules.^25,27,28^ It has been proven that such structural motifs can be responsible for the metallic and accompanying superconducting character of hydrides.^31,32^ The electronic properties of both BaH_4_ and Ba_8_H_46_ have not been previously studied.

We have conducted a comprehensive study on how compression influences the transport properties of BaH_2_ and how these are altered by additional hydrogen uptake. We combine optical spectroscopy and X-ray diffraction with electrical transport measurements to investigate the behavior of BaH_2_ and Ba_8_H_46_ upon compression. X-ray diffraction confirms the BaH_2_-II (Ni_2_In type) → BaH_2_-III (SH) transition at 53 GPa, which is supported by changes in the Raman spectra. We observe that the sample becomes reflective above pressures of 53 GPa, while the extrapolation of the absorbance curve shows a pressure-induced band gap closure at 55(5) GPa. Our electrical resistance measurements confirm an insulator–metal transition undergoing completion by 57 GPa in BaH_2_. Electrical transport measurements performed on Ba_8_H_46_, synthesized from BaH_2_ + H_2_ at 45 GPa and 1200 K, show that the compound is metallic upon a pressure increase to 85 GPa.

For a detailed description of the experimental methods, the reader is referred to the Supporting Information. The BaH_2_ sample was loaded and compressed to 13 GPa (surpassing the BaH_2_-I → BaH_2_-II transition). BaH_2_-II has two active E_g_ vibrational Raman modes: one at 105 cm^–1^, associated with the vibrational modes of the Ba atoms, and a broader excitation at 1134 cm^–1^, assigned to the vibrational mode of the hydrogen atoms^21,23^ (Figure 1a). With compression, the higher wavenumber mode hardens until it overlaps with the diamond mode at 34 GPa. At 53 GPa, the low-frequency mode related to Ba phonons decreases in intensity and disappears. These spectral changes in the Raman signature are consistent with previous studies.^21,23^ X-ray diffraction data were collected from 14 to 62 GPa at 300 K (Figure 1b) and are also in agreement with previous works.^21−23,29^ We observe that, at 14 GPa, a Ni_2_In-type hexagonal close-packed structure is adopted by BaH_2_-II. Upon further compression, at 35 GPa, we find additional diffraction peaks that can be indexed to a simple hexagonal (SH) structure corresponding to BaH_2_-III, emerging together with BaH_2_-II (Ni_2_In type). This overlap of phases remains up to a pressure of 47 GPa. Beyond this pressure, only BaH_2_-III (SH) is observed in the diffraction pattern up to 62 GPa.

**Figure 1:**
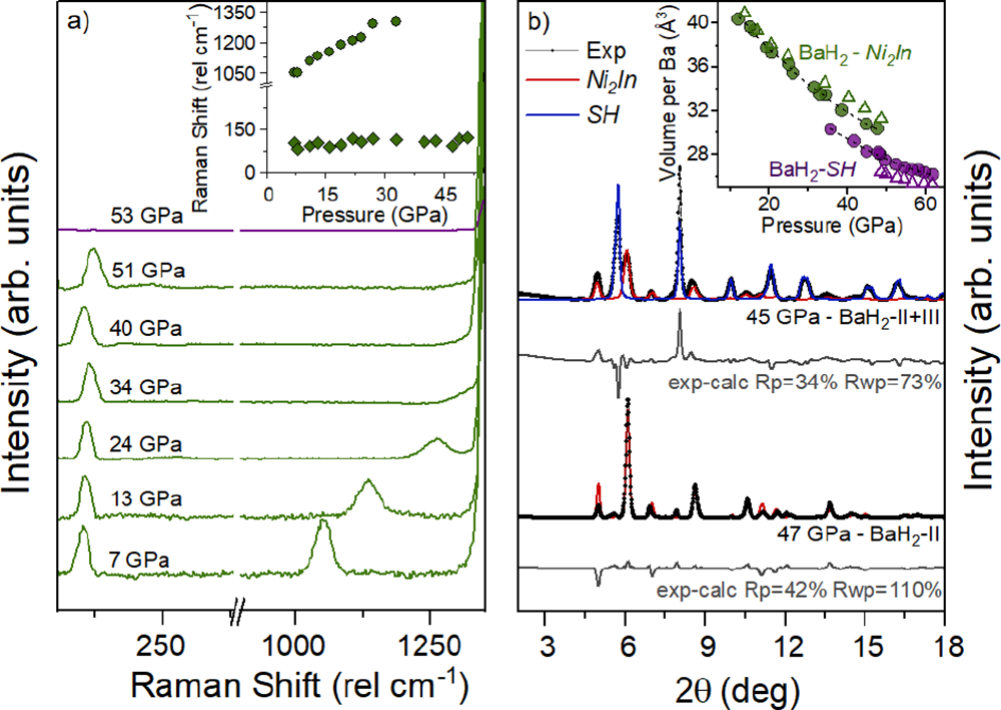
(a) Raman spectra on compression of BaH_2_-II (green) and BaH_2_-III (purple). The inset displays the Raman shift as a function of pressure for BaH_2_. The low-frequency data marked with diamonds correspond to Ba–Ba phonons, while the high-frequency values presented as circles correspond to the vibrational modes of the hydrogen atoms.^21,23^ (b) Rietveld refinements of BaH_2_-II at 47 GPa (Ni_2_In-type hexagonal close-packed structure with *a* = 4.1630 Å and *c* = 5.2886 Å) and BaH_2_-II + BaH_2_-III at 45 GPa (SH structure with *a* = 3.3648 Å and *c* = 2.9727 Å). A wavelength of λ = 0.2922 Å was used. The inset displays the volume per Ba atom as a function of pressure, with green symbols representing BaH_2_-II and purple symbols representing BaH_2_-III. Circular symbols correspond to our data, while empty triangles denote previously published volumetric data.^22^

To investigate any pressure-induced changes to the electronic properties of BaH_2_, we measured the absorption and reflectivity between 1.2 and 2.8 eV. At ambient conditions, BaH_2_ is a white, transparent solid with negligible reflectance when compared to gold (Figure 2b). With compression, the sample visibly darkens (Figure 2a), accompanied by a rapid increase in the reflectivity. For example, at 13 GPa, the reflectance at 2.1 eV is 38% when compared to gold, and at 19 GPa, this increases to 81%. At 40 GPa, the reflectance of the sample surpasses that of the rhenium gasket. At 53 GPa, the sample becomes almost entirely reflective compared to gold, with a maximum reflectance value of 98% at 2.1 eV.

**Figure 2:**
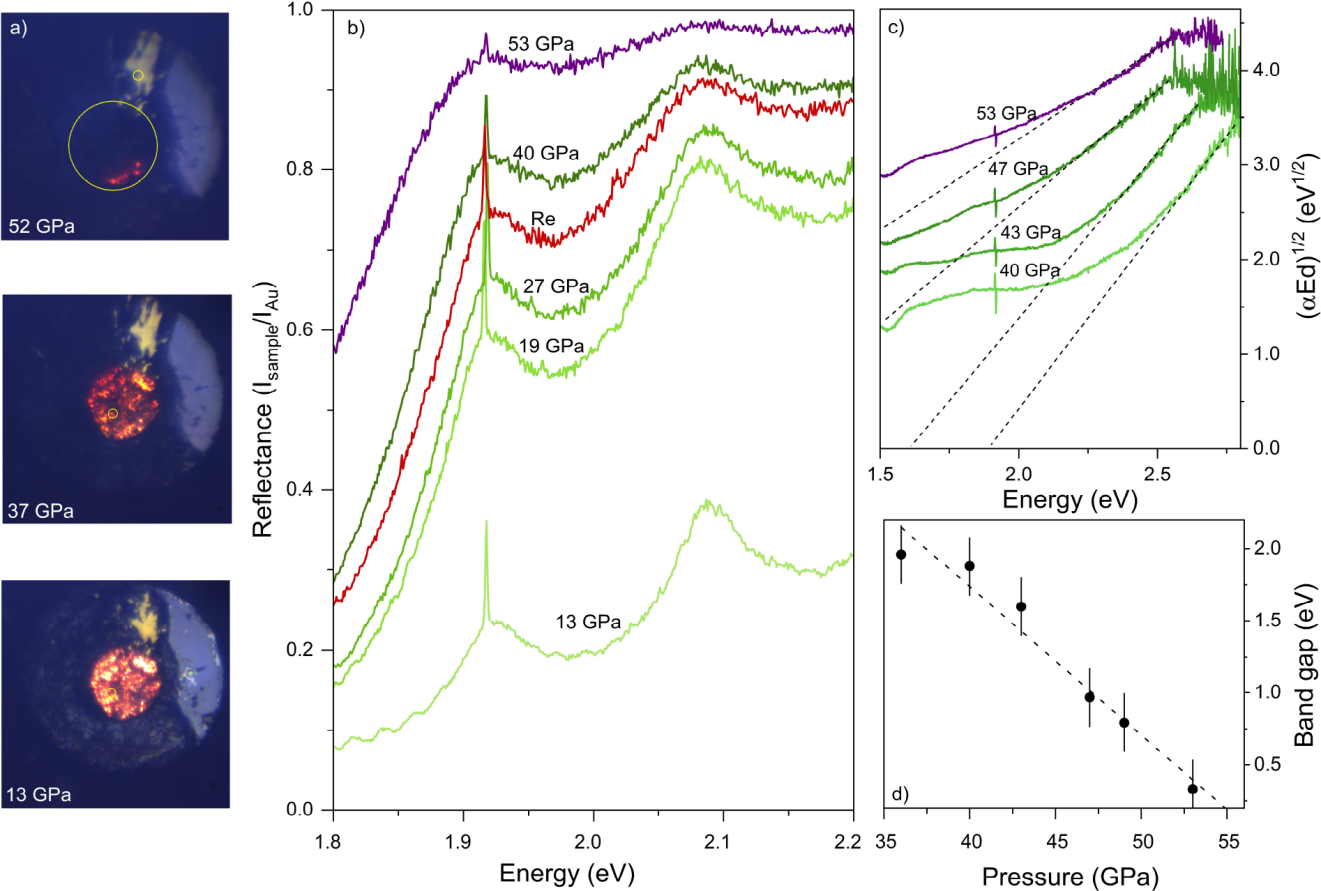
(a) Photomicrographs of BaH_2_, taken with transmitted and reflected light, at 13, 37, and 52 GPa. Gold foil can be observed on the gasket, which is used as a reference for reflectivity measurements. (b) Representative reflectivity data from a supercontinuum source at various pressures relative to a gold reference, where *I*_sample_ is the sample–diamond interface and *I*_au_ is the gold–diamond interface. The reflectance spectrum of rhenium is displayed in red for comparison. The peaks at 1.91 eV are due to the notch filter. (c) Representavie absorbance spectra (*A*) of BaH_2_ from a broadband white light source presented as α*Ed*^0.5^ versus energy, where α is the absorption coefficient and *d* is the sample thickness, the α*d* = *A* ln_10_ relationship is used. Linear extrapolation of the absorbance data gives an approximate value of the band gap.^33−35^ (d) Approximate electronic band gap (E_g_) of BaH_2_ as a function of the pressure. A linear fit of these values gives complete band gap closure at 55(5) GPa.

The absorption (*A*) measurements are in excellent agreement with the reflectivity. Below 24 GPa, the sample is almost entirely transparent (Figure S1 of the Supporting Information). Upon compression to 36 GPa, the sample visibly darkened and the absorbance increased (Figure S2 of the Supporting Information). In Figure 2c, we present the absorption data plotted in photon energy versus α*Ed*^0.5^ (where α is the absorption coefficient, *d* is the sample thickness, and α*d* = *A* ln_10_). The absorbance continuously increases upon further compression, reaching its maximum at 53 GPa, which is the highest pressure reached. Linear extrapolation of the absorption edge allows for the estimation of the band gap, which, at 40 GPa, is approximately 1.9(2) eV (Figure 2d). At 53 GPa, the sample shows virtually no transmission within the visible region. An approximately linear decrease in the band gap is seen through extrapolation of the absorbance edge. This gives a calculated band gap closure at an estimated pressure of 55(5) GPa.

Although optical measurements are highly indicative of metallicity, electrical transport measurements provide definitive insights into the electronic character of a given system. Thus, we conducted electrical resistance measurements on BaH_2_ up to 60 GPa (Figure 3a). Both direct current (DC) and alternating current (AC) resistance measurements as a function of the pressure were carried out in both two- and four-probe configurations (Figure S6 of the Supporting Information). At ambient conditions, BaH_2_ behaves as an insulating material. A measurable resistance of 100 MΩ appears only at a pressure of 21 GPa. Because such a large magnitude of resistance was measured across the semiconducting sample, impedance spectroscopy was implemented (Figure S3 of the Supporting Information) to eliminate contributions from inductive and capacitive effects. The reader is referred to the Supporting Information for further details. Between 20 and 35 GPa, the resistance only slightly decreases, changing from 6.2 MΩ at 21 GPa to 4.8 MΩ at 35 GPa, as highlighted in Figure 3a. Further compression beyond 35 GPa leads to a sharp decrease in resistance, dropping to 2.2 MΩ at 37 GPa. This is in agreement with the observations from our X-ray diffraction measurements, where the onset of the transformation into BaH_2_-III begins at 35 GPa, resulting in the reduction in the average band gap of the bulk sample. Above 57 GPa, the resistance of the sample reaches mΩ values and begins to plateau, indicating complete metallization of the bulk sample.

**Figure 3:**
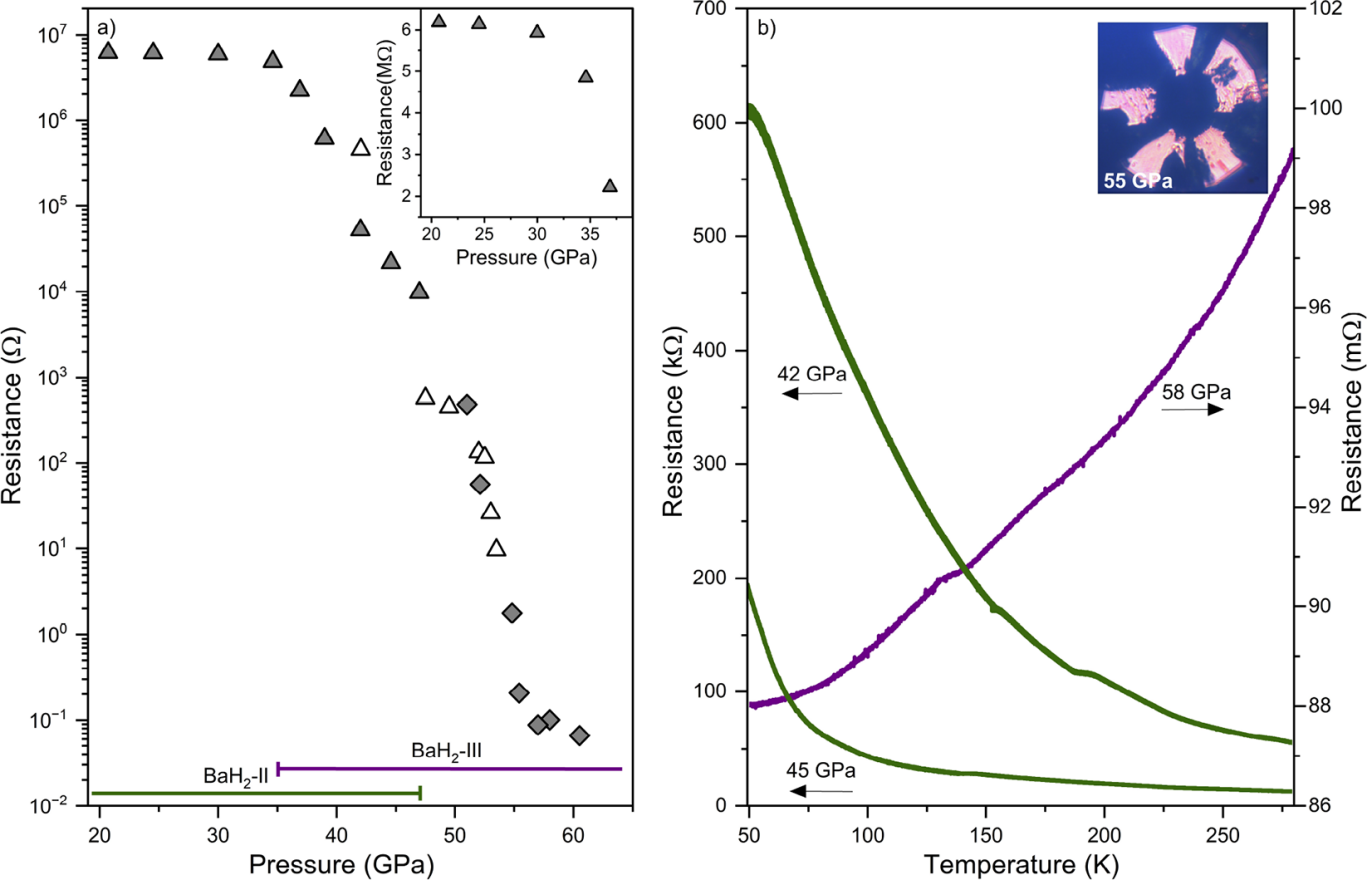
(a) Resistance as a function of the pressure for BaH_2_ at ambient temperature in between 21 and 60 GPa. Solid symbols indicate AC measurements, while the empty symbols show DC measurements. Two-probe resistance measurements are shown with triangles, while diamonds are from the four-probe runs. The pressure regions where BaH_2_-II and BaH_2_-III are present in the X-ray diffraction data are indicated. The inset displays the resistance as a function of the pressure for BaH_2_ between 21 and 32 GPa, highlighting that, in the low-pressure regime, resistance still decreases. (b) Resistance of BaH_2_ as a function of the temperature at 42, 45 and 58 GPa, carried out on the same sample. The green data correspond to semiconducting BaH_2_-II measured in a two-probe configuration using an AC and are linked to the left *y* axis. The purple data correspond to metallic BaH_2_-III measured using the four-probe method with an AC and are linked to the right *y* axis. The inset displays the photomicrograph of BaH_2_ at 52 GPa, taken with transmitted and reflected light.

We conducted temperature-dependent resistance measurements of BaH_2_ at various pressures. At 42 GPa, as shown in Figure 3b, BaH_2_ exhibits increasing resistance upon cooling. This is a characteristic of semiconducting materials, with fewer charge carriers able to be thermally promoted to the conduction band as the sample is cooled. Upon further compression to 45 GPa, the resistance as a function of the temperature still displays semiconducting behavior. This dependency changes at 58 GPa, at which the resistance decreases with cooling, distinctive of metallic behavior. Such a turnover shows a semiconductor–metal transition between 47 and 58 GPa. This is attributed to the s → d hybridization of the Ba atom and a charge transfer from H^–^ to the metallic ion.^20−22^

To evaluate the effect that incorporating hydrogen into BaH_2_ would have on its electrical properties, a sample of BaH_2_ was laser-heated in a dense hydrogen matrix. Because BaH_4_ has distinctive Raman modes^25^ and the Raman spectrum of Ba_8_H_46_ is featureless,^27^ we implement Raman spectroscopy as in-house diagnostics for their synthesis, following previously established synthesis routes. Upon compression of BaH_2_ and H_2_ to 38 GPa, BaH_4_ forms (Figure 4a), which is in agreement with previous results.^25^ This is characterized by the growth of a broad mode at 3400 cm^–1^ related to the H–H intramolecular stretching of the H_2_ vibrational mode (vibron). Additionally, modes around 1000 and 1250 cm^–1^ from hindered rotational modes (rotons) of BaH_4_^25^ are seen in the Raman spectrum, along with low-frequency Ba–Ba phonons. Upon laser heating the sample at 45 GPa to a temperature of 1200 K, the Raman spectrum resembles that previously reported for Ba_8_H_46_,^27,28^ in which the low-frequency Ba–Ba phonons and H–H stretching modes of BaH_4_ are no longer resolvable. Along with this, the intensity of the H_2_ vibron decreases, suggesting that the amount of free molecular hydrogen in the sample chamber is reduced. X-ray diffraction was implemented to structurally characterize the synthesis product at 45 GPa (Figure S4 of the Supporting Information).

**Figure 4:**
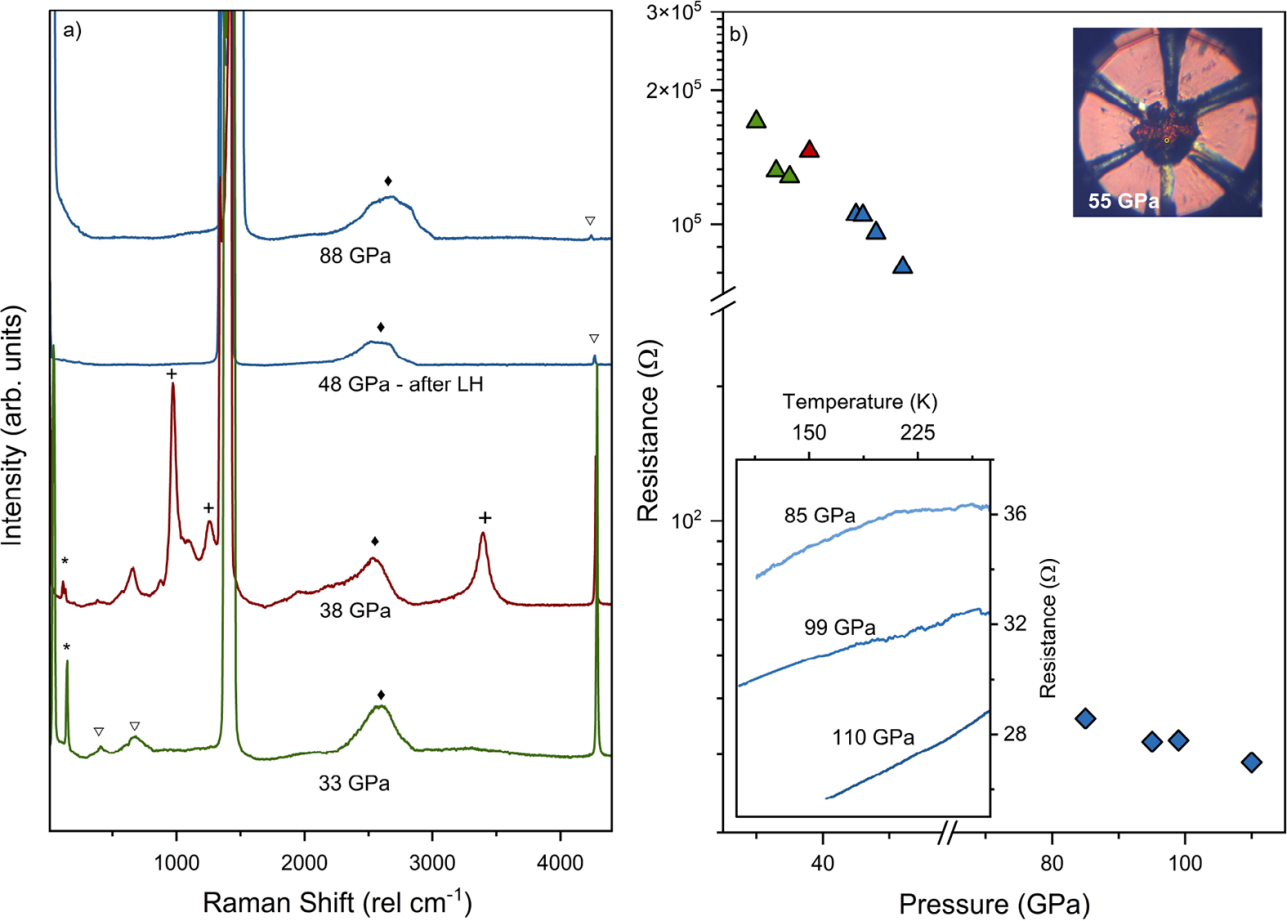
(a) Raman spectra of BaH_2_ + H_2_ at 33 GPa (green), BaH_4_ formed at 38 GPa (red), and Ba_8_H_46_ (blue). The characteristic modes of BaH_4_ are marked with crosses, while empty triangles mark the rotational modes and vibron of pure hydrogen. A diamond marks the second-order diamond Raman mode, and an asterisk denotes the Ba–Ba phonon. (b) Resistance of BaH_2_ + H_2_ (green), BaH_4_ (red), and Ba_8_H_46_ (blue) as a function of the pressure. The top inset displays the photomicrograph of Ba_8_H_46_ at 55 GPa, taken with transmitted and reflected light. The bottom inset displays the resistance as a function of the temperature of Ba_8_H_46_ at 85, 99, and 110 GPa.

We measured the pressure-dependent resistance during the synthesis process from BaH_2_ + H_2_ → BaH_4_ → Ba_8_H_46_. Impedance spectroscopy was also implemented (Figure S5 of the Supporting Information). Upon BaH_4_ synthesis, there is an increase in resistance from 128 to 146 kΩ. Unlike BaH_2_, BaH_4_ contains molecular hydrogen.^28^ The electronic structure of BaH_4_ has distinct bands associated with Ba *5p*, molecular H_2_, and atomic hydrogen, which gives a band structure of a small-gap semiconductor.^28^ Therefore, the experimentally observed increase in resistance when BaH_4_ forms is likely due to the presence of molecular hydrogen in the structure.^25,28^ Upon the formation of Ba_8_H_46_ at 45 GPa, the resistance slightly drops to 105 kΩ. Compression of Ba_8_H_46_ at 55 GPa leads to a decrease in resistance to 48 kΩ. With the sample still transmitting light (Figure 4b), it is evident that the band gap closure has not yet occurred at 55 GPa. Further compression to 85 GPa results in a significant and rapid decrease in resistance to 36 Ω, approaching values typically associated with a metallic phase. This is accompanied by the sample darkening and becoming reflective (Figure S6 of the Supporting Information). At pressures above 85 GPa, the sample resistance plateaus. These results indicate a band gap closure of Ba_8_H_46_ by 85 GPa. At 85, 99, and 110 GPa, Figure 4b shows that the resistance of Ba_8_H_46_ drops during cooling, exhibiting metallic behavior. This confirms that the metallic transition occurs by 85 GPa, a pressure significantly higher than that required for BaH_2_.

Since it was proposed by Ashcroft that chemical pre-compression of hydrogen could be achieved by doping with electropositive elements, it has been debated whether a higher hydrogen content will result in a higher superconducting critical temperature.^19^ Understanding the electronic properties as a function of the hydrogen content is important in addressing this problem. Here, we highlight how hydrogen incorporation in the Ba–H system increases the pressure required for metallization. Our results leave open the potential to expand studies of metallization pressures in the Ba–H system up to BaH_12_, which is a superconductor with an experimentally measured *T*_c_ of 20 K at 140 GPa.^11^ Our findings can be extended to other heavy alkaline earth hydrides, such as Ca–H. Metallization in CaH_2_ has not been experimentally measured, although it has been predicted to occur at 102 GPa,^20^ while CaH_6_ has been experimentally observed to become metallic and superconducting over 170 GPa, upshifted from that predicted for CaH_2_.^15,16^ While not the only factor, hydrogen incorporation in such systems is correlated with an increase in the critical temperature of superconductivity. However, the drawback is that higher and more experimentally challenging pressures are required.

In summary, the insulator–metal transition in BaH_2_ has been thoroughly characterized using a series of optical measurements in the visible range and electrical resistance measurements, in addition to Raman spectroscopy and X-ray diffraction for structural assignment. Absorbance measurements allow us to estimate an approximate band gap of 1.9(2) eV at 40 GPa, a value typical for semiconducting materials.^33^ Completion of sluggish BaH_2_-II (Ni_2_In type) → BaH_2_-III (SH) at 54 GPa produces the complete closure of the average bulk sample electronic band gap, a characteristic of a metallic state. Electrical transport measurements as a function of the pressure and temperature confirm the insulator–metal transition in BaH_2_ at 57 GPa. We demonstrate that the incorporation of H_2_ into BaH_2_ to form Ba_8_H_46_ results in an upshift in the pressure of metallization to 85 GPa.

## Supporting Information Available

Experimental methods, raw transmission data and absorbance spectra, Rietveld refinement of Ba_8_H_46_, and impedance spectroscopy measurements (PDF)
Transparent Peer Review report available (PDF)
