# Supplementary material for: Pressure-Induced Metallization of BaH2 and the Effect of Hydrogenation
Source: J Phys Chem Lett. 2023 Dec 12;14(50):11490–6. doi: 10.1021/acs.jpclett.3c02704 (PMC10749470; doi:10.1021/acs.jpclett.3c02704)
Supplement: Supplementary file 1 — jz3c02704_si_001.pdf [file jz3c02704_si_001.pdf]

Supporting Information: "Pressure-Induced Metallization of BaH_2 and the Effect of Hydrogenation"

Hannah A. Shuttleworth,[†] Israel Osmond,[†] Calum Strain,[†] Jack Binns,[‡] Jonathan Buhot,[¶] Sven Friedemann,[¶] Ross T. Howie,[†,‡] Eugene Gregoryanz,[*,†,‡,§] and Miriam Peña-Alvarez[†]

†*Centre for Science at Extreme Conditions, the University of Edinburgh, Edinburgh, EH8 8AQ, U.K.*

‡*Center for High Pressure Science and Technology Advanced Research, 1690 Cailun Road, Shanghai, 201203, China*

¶*H.H. Wills Physics Laboratory, University of Bristol, Bristol BS8 1TL, U.K.*

§*Key Laboratory of Materials Physics, Institute of Solid State Physics, CAS, Hefei, China*

E-mail: e.gregoryanz@ed.ac.uk

Raman spectroscopy and X-ray Diffraction Measurements of BaH_2

To obtain Raman scattering and X-ray diffraction data on BaH_2, a sample of 99.5% purity ABCR BaH_2 was loaded into a diamond anvil cell (DAC) in an argon atmosphere glovebox, along with gold. Pressures were generated using symmetric diamond-anvil cells of our own design equipped with diamonds having culet sizes of 200 µm. Rhenium foils of 200-250 µm thick were used as gasket material. Sample purity was thoroughly checked after loading BaH_2 by Raman spectroscopy. Pressures were determined through comparison to previously reported Raman spectroscopy data[1,2] and the diamond Raman scale.[3] Raman spectroscopy measurements were conducted using 532 and 514.5 nm excitation wavelengths via a custom-built micro-focused Raman system provided with 1800 gr/mm and 300 gr/mm gratings and a charge coupling device (CCD) array detector. X-ray diffraction data were collected at P02.2 Petra-III, Germany. Intensity vs. 2θ plots were obtained by integrating image plate data in various formats using DIOPTAS.[4] Rietveld refinements were carried out in PowderCell.[5,6]

Transmission and Reflection Measurements of BaH_2

Measurements were obtained by loading a sample of BaH_2 in a similar manner, with gold clamped between the gasket and the diamond culet. Gold was used to normalize reflection measurements. Reflectivity measurements were performed using a Leukos STM4 supercontinuum source. Transmission measurements were done using a broadband white light source. Both types of measurements were couple to a custom-built Raman system provided with 150 gr/mm grating and a charge coupling device (CCD) array detector to determine pressure, with an excitation wavelength of 647 nm. The transmission was normalized by the transmission spectrum of the empty cell before loading.

Electrical Measurements of BaH_2

Electrodes were created on the 200 μm diamond culets by manually placing hand-cut platinum foil electrodes. Five electrodes were placed as a precaution, in case of electrode(s) failing during compression. An insulating gasket was made from a mixture of Al_2O_3 and

epoxy mixture. DC measurements were carried out a Keithley 2002 Bench Digital Multimeter. AC measurements were carried out using a Keithley 2400 Series Source Meter in combination with an R830 (Stanford Research Systems) lock-in amplifier for alternating current measurements. With a semiconducting sample, four-probe measurements are not necessary, as the sample resistance is significantly larger than the contact and lead resistance. Two-probe measurements were carried out with an excitation current of 100 nA applied between contacts B and C, shown in the left panel of Figure S6. Impedance spectroscopy was implemented when the sample was in a semiconducting state, in order to eliminate contributions from inductive and capacitive effects. Additionally, this allows the determination of the optimum reference frequency for measurements, ensuring that measurements are consistent throughout compression. The sample resistance is calculated from the value of Re(V) where Im(V) $\rightarrow 0$ and $\omega \sim 3$ Hz, i.e. where capacitive and inductive effects are negligible. At 39.8 GPa, Re(V) showed little change with varying ω and impedance spectroscopy was no longer required. Above this pressure, resistance measurements were taken at a constant reference frequency of 3 Hz. At 47 GPa, the excitation current was increased to 10 μA. Cooling runs at 42 and 45 GPa were also carried out with these parameters: 10 μA and 3 Hz in a two-probe configuration. At 51 GPa, a four-probe configuration was used to eliminate contact and lead resistance, with an excitation current of 10 μA applied across electrodes A-D and voltage measured across B-C. Four-probe measurements were continually checked against two-probe measurements. The cooling run at 58 GPa was carried out with the four-probe configuration.

Electrical Measurements and Synthesis of Ba_8H_{46}

For the electrical measurements of Ba_8H_{46}, two cells were prepared, one using diamonds with 200 µm culets and the other with 100 µm. For each cell, thin-film electrodes of tungsten and gold were deposited on one of the culets and then they were reinforced with hand-placed Pt foil at the culet edge. Both DACs were loaded with BaH_2 in an inert atmosphere, before opening and closing the cell within a 2 kbar research-grade hydrogen (99.9995%) atmosphere

to provide both the reactant and a hydrostatic pressure transmitting medium. To synthesize BaH_4, BaH_2+H_2 was heated *in situ* by directly coupling to a yttrium-aluminum-garnet (YAG) continuous wave laser with wavelength λ=1064 nm. We estimate temperatures of 1200 ± 50 K from the detected black-body radiation. For the 200 µm culet DAC, impedance spectroscopy curves were measured upon compression with a 1 µA excitation current between contacts B and C, shown in the middle panel of Figure S6. For measurements above 45 GPa, four-probe AC resistance measurements were performed with a 100 µA excitation current with current applied between contacts A-D and voltage measured between contacts B-C, shown in the right panel of Figure S6. A reference frequency of 3 Hz was used.

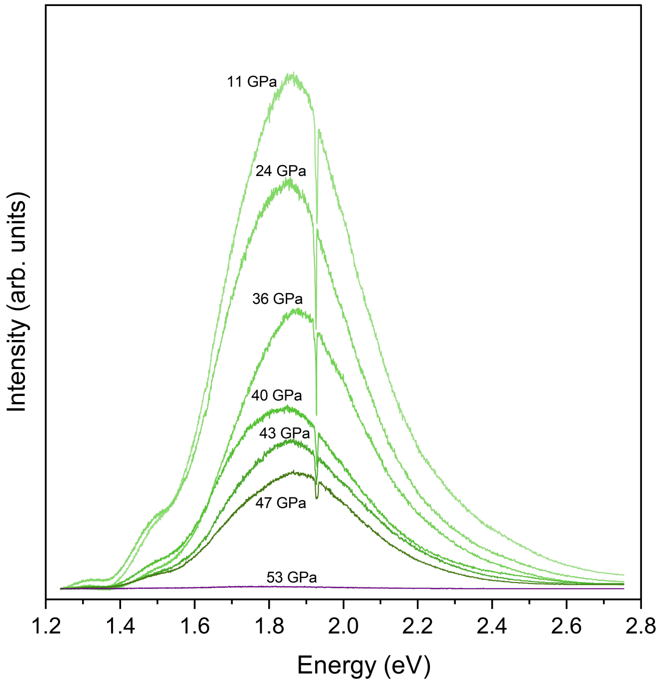

Figure S1: Raw transmission data of BaH_2. The peaks at 1.91 eV are due to the notch filters.

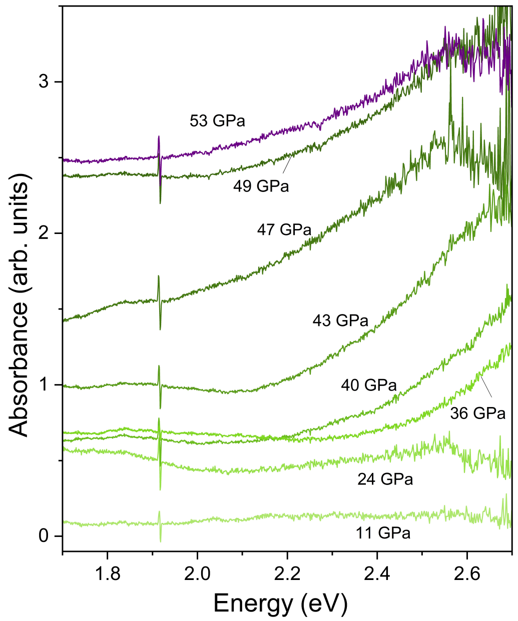

Figure S2: Absorbance spectra for BaH_2 up to 53 GPa calculated from the transmission spectra. Up to 24 GPa, absorbance is low. The absorbance reaches a maximum value at 53 GPa.

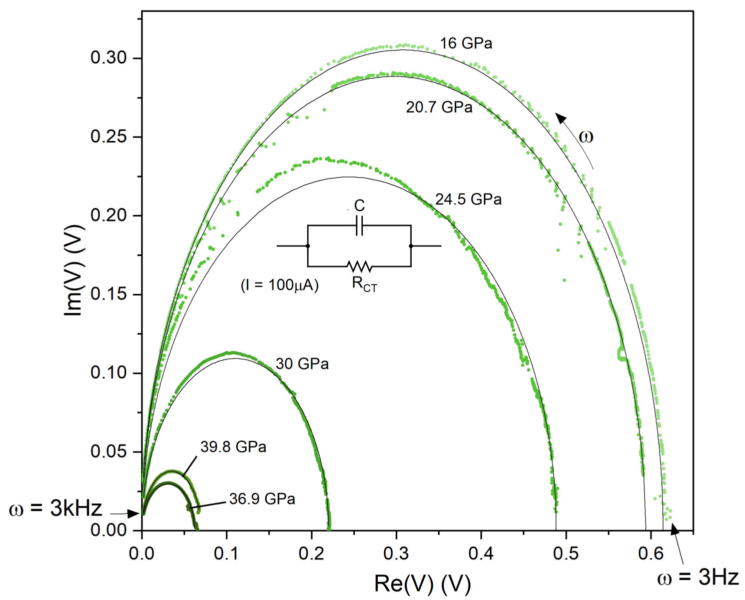

Figure S3: Impedance spectra of BaH_2 up to 36.9 GPa. Here, R_{CT} denotes the charge transfer resistance of the interface, i.e. the sample resistance. The sample resistance in Figure 3a is calculated from Re(V) where Im(V) $\rightarrow$ 0 and $\omega \sim 3$ Hz. Beyond 36.9 GPa, impedance becomes negligible, so AC measurements were carried out at a fixed reference frequency of 3 Hz. Sample resistance values are taken to be the value at ω=0, which the impedance spectra are extrapolated to. A current of 100nA was used.

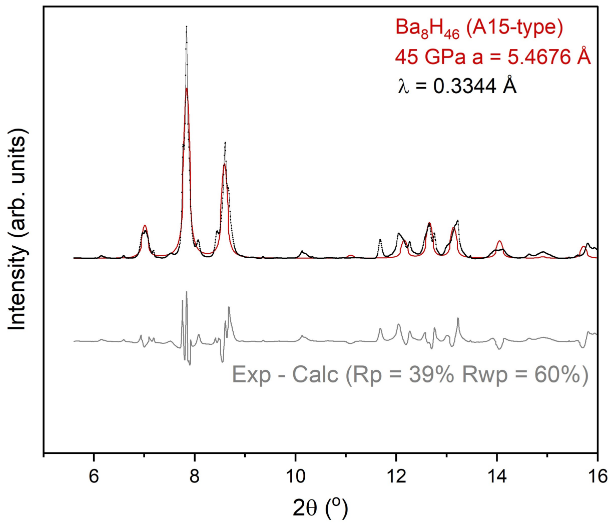

Figure S4: Rietveld refinements of Ba_8H_{46} at 45 GPa (*Pm-3n* with a = 5.4676 Å), used to structurally characterize the product of laser heating BaH_4 + H_2. A wavelength of λ = 0.3344 Å was used.

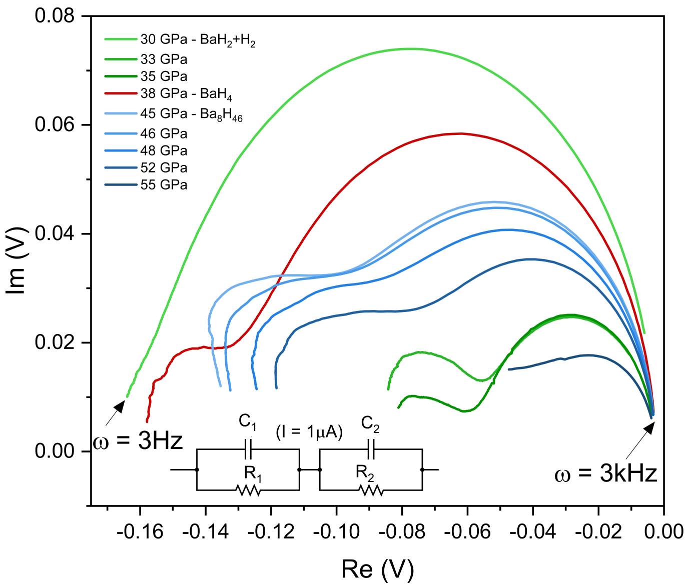

Figure S5: Impedance spectra of $BaH_2 \rightarrow BaH_4 \rightarrow Ba_8H_{46}$ up to 55 GPa. Here, R_1 denotes the charge transfer resistance of the interface, i.e. the sample resistance. R_2 denotes the electrode resistance. Sample resistance values are taken to be the value at ω=0, which the impedance spectra are extrapolated to. The sample resistance is calculated from Re(V) where Im(V) $\rightarrow$ 0 and $\omega \sim 3$ Hz. A current of 1μA was used.

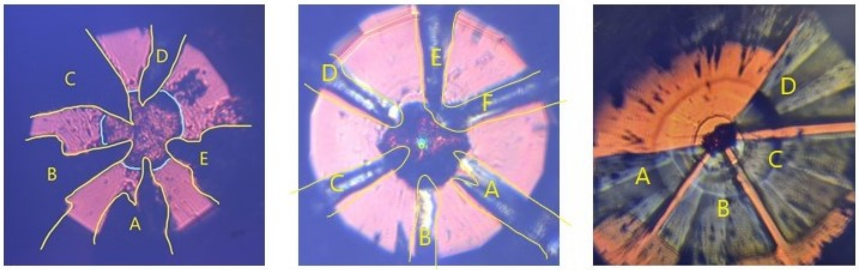

Figure S6: Photomicrographs of the electrode configuration used for electrical transport measurements. Left: BaH_2 at 38 GPa, with 200 μm culets. Middle: Ba_8H_{46} at 55 GPa. This loading was used for electrical measurements up to 55 GPa, with 200 μm culets. Right: Ba_8H_{46} at 85 GPa. This loading was used for electrical measurements beyond 55 GPa, with 100 μm culets.

References

(1) Smith, J. S.; Desgreniers, S.; Tse, J. S.; Klug, D. D. High-pressure phase transition observed in barium hydride. *Journal of Applied Physics* **2007**, *102*, 043520.

(2) Tse, J. S.; Song, Z.; Yao, Y.; Smith, J. S.; Desgreniers, S.; Klug, D. D. Structure and electronic properties of BaH_2 at high pressure. *Solid State Communications* **2009**, *149*, 1944–1946.

(3) Akahama, Y.; Kawamura, H. Pressure calibration of diamond anvil Raman gauge to 310 GPa. *Journal of Applied Physics* **2006**, *100*.

(4) Prescher, C.; Prakapenka, V. B. DIOPTAS: a program for reduction of two-dimensional X-ray diffraction data and data exploration. *High Pressure Research* **2015**, *35*, 223–230.

(5) Rietveld, H. M. A profile refinement method for nuclear and magnetic structures. *Journal of Applied Crystallography* **1969**, *2*, 65–71.

(6) Kraus, W.; Nolze, G. POWDER CELL–a program for the representation and manipulation of crystal structures and calculation of the resulting X-ray powder patterns. *Journal of Applied Crystallography* **1996**, *29*, 301–303.
