# Supplementary material for: Pressure-Induced Metallization of BaH2 and the Effect of Hydrogenation
Source: J Phys Chem Lett. 2023 Dec 12;14(50):11490–6. doi: 10.1021/acs.jpclett.3c02704 (PMC10749470; doi:10.1021/acs.jpclett.3c02704)
Supplement: Supplementary file 2 — jz3c02704_si_002.pdf [file jz3c02704_si_002.pdf]

jz-2023-02704c.R1

Name: Peer Review Information for "Pressure-Induced Metallization of BaH₂ and the Effect of Hydrogenation"

First Round of Reviewer Comments

Reviewer: 1

Comments to the Author

In this work, the authors present a study on pressure-induced metallization in BaH2 and Ba8H46 using optical spectroscopy, X-ray diffraction and electrical transport measurements. Overall, the study is serious and the results are interesting and I do really recommend their publication in "The Journal of Physical Chemistry Letters". Nevertheless, the manuscript needs some major revisions which the authors need to consider prior to publication.

Q1. The authors mentioned the simple hexagonal (SH) structure of BaH2-III, emerging together with BaH2-II (Ni2In-type) up to 47 GPa. However, the figure 1.b shows only the SH phase at 45 GPa and Ni2ln phase at 47 GPa. The XRD patterns should contain mix phases in this pressure range?

Q2. In figure 1.a, higher wave-number mode which assigned to the hydrogen atoms vibrational mode disappear above 34 GPa， does this relate to the structure transition of BaH2 under high pressure？

Q3. Does the Raman and XRD experiments carried out in hydrostatic environment? Different pressure environments can affect the pressure of metallization.

Q4. From the insert pictures in fig3 and fig4, the authors seem to use six probes to carry out the resistance measurement. Why using this configuration?

Q5. The authors mentioned ‘the predicted metallization pressure is 102GPa (Tc = 1 mK) for CaH2, 177 GPa for SrH2 and 50 GPa for BaH2 (Tc = 13 mK). Can signature of superconductivity be found in BaH2 above 50GPa?

Q6. The authors used Raman spectroscopy as in-house diagnostics for both BaH_4 and Ba_8H_{46}. However, Raman spectrum of Ba_8H_{46} is featureless. The product of BaH_4 + H_2 after laser heating still remain unclear. Further analysis such as XRD should be carried out to confirm the structure.

Q7. Resistance of the metallic BaH_2 reaches mΩ values, yet, the resistance of as claimed metallic Ba_8H_{46} plateaus around 36 Ω. What leads to this huge difference?

Q8. The resistance-temperature relation presented in fig4 suggests the sample is not well connected. Does the laser-heated sample in mixed phases above 85 GPa?

Reviewer: 2

Comments to the Author

The manuscript contains interesting structural and transport studies of barium dihydride under high-pressure conditions. Structural studies confirm the previously published theoretical and experimental information on pressure-induced phase transitions and changes in the stoichiometry of barium hydrides. Significantly new are only the measurements of barium hydrogen resistance and the insulator-metal transition under the influence of high pressure, also documented by optical and X measurements. The search for high-temperature superconductivity in metal hydrides has recently become widespread, and the technique of measuring electrical resistance is becoming a routine technique in many laboratories.

In my opinion, the topics and methods used in the research presented in the manuscript are not particularly innovative and the results were easy to predict in the light of existing knowledge. Nevertheless, the observation of the insulator-metal transition will probably be interesting for many researchers from the point of view of the electronic structure of metal-hydrogen systems.

I have a few comments regarding the structure of the manuscript itself.

1. The influence of hydrostatic pressure on the properties of various materials is documented by hundreds of publications, and their selection as references should strictly concern the methodology and subject matter of the presented research. Therefore, works on high-pressure properties of materials such as silane, sulfur, or nitrogen should not be cited. There are enough works on high-pressure hydride research to place our research there.

2. In many cases, linking high-pressure research on hydrides with their use as hydrogen or energy storage materials is irrational. This also applies to barium hydride with a hydrogen content of less than 1.5 percent by weight and high thermodynamic stability. The cited works 14-16 do not confirm the possibility of using alkaline earth hydrides as hydrogen stores. Theoretical paper 14 in Conclusions states in its only sentence that these hydrides have the potential as hydrogen storage facilities without any justification. Paper 15 only states that magnesium hydride, despite its high hydrogen content, is not suitable for storage due to the thermodynamic properties of this hydride. The same information can be

found in paper 16 regarding calcium hydride. Mindless repetition of populist information addressed mainly to funders and having no scientific basis is not appropriate for authors who aspire to have their works published in serious scientific journals.

3. As for the References, paper 24 has no authors (sic!).

4. There is no information about the origin and purity of the hydride used.

5. Some drawings presenting the research results are difficult to read or require supplementation

a) Fig. 1 smaller triangles denote previously published volumetric data that are invisible even at 5x magnification.

b) Fig. 3 b, it is not clear whether the data presented concern the same sample or different measurements. Is there no data regarding pressures between 42 and 58 GPa? They would be interesting for a more accurate determination of the insulator-metal transition pressure.

6. It can be noticed that the Authors prefer to cite their works. This applies, for example, to papers 36 and 37. Optical methods for determining band gap closure have been used many times in older publications and the first ones should be cited instead. For example see “High-pressure study of ScH_3: Raman, infrared, and visible absorption spectroscopy, Tetsuji Kume, Hiroyuki Ohura, Tomoo Takeichi, Ayako Ohmura, Akihiko Machida, Tetsu Watanuki, Katsutoshi Aoki, Shigeo Sasaki, Hiroyasu Shimizu, and Kenichi Takemura, Phys. Rev. B 84, 064132, 2011, and references given there.

7. In several places the authors use the term insulator-metallic transition instead of insulator-metal

8. In the sentence on Page 10, lines 48,49 “by” is probably missing.

Taking into account the above comments regarding the innovativeness of the work and its presentation, I cannot recommend the manuscript for publication in the Journal of Physical Chemistry Letters in the present form. I suggest that, after taking into account my comments, the manuscript should be submitted to the Journal of Phys, Chem. C were it will be more suitable regarding the scientific level.

Author's Response to Peer Review Comments:

Reply to the Referees on "Pressure-Induced Metallization of BaH_2 and the Effect of Hydrogenation", H. A. Shuttleworth *et al.*

October 20, 2023

We would like to thank all referees for providing useful and critical assessments of our work. We have answered all questions posed by the referees and made the necessary corrections. Please find our point-by-point response below to the referee reports. The referees' comments are in blue italics and our response follows in normal font.

Reviewer: 1

Recommendation: This paper may be publishable, but major revision is needed; I would like to be invited to review any future revisions.

Comments: In this work, the authors present a study on pressure-induced metallization in BaH_2 and Ba_8H_{46} using optical spectroscopy, X-ray diffraction and electrical transport measurements. Overall, the study is serious and the results are interesting and I do really recommend their publication in "The Journal of Physical Chemistry Letters". Nevertheless, the manuscript needs some major revisions which the authors need to consider prior to publication.

We thank the referee for praising our work and finding our results serious and interesting. Below we are answering the questions posed by the referee, indicating the changes we have made to the paper.

Q1. *The authors mentioned the simple hexagonal (SH) structure of BaH2-III, emerging together with BaH_2-II (Ni_2In-type) up to 47 GPa. However, the figure 1.b shows only the SH phase at 45 GPa and Ni_2In phase at 47 GPa. The XRD patterns should contain mix phases in this pressure range?*

We thank the reviewer for point this out. During the given pressure range (35 - 47 GPa), regions of the sample adopted a pure Ni_2In-type hcp structure, some a pure simple hexagonal and some a mixed phase. We have made changes to Figure 1b to clarify this. Now, Rietveld refinements of BaH_2-II + BaH_2+III at 45 GPa are given, displaying the mixed phase.

Q2. In figure 1.a, higher wave-number mode which assigned to the hydrogen atoms vibrational mode disappear above 34 GPadoes this relate to the structure transition of BaH_2 under high pressure

It could be, but X-ray diffraction shows that BaH_2-II (Ni_2In) persists up to 47 GPa. The irreducible representation of the Raman modes for this phase is $\Gamma_{\text{Raman}} = 2E_g$ (Ref. 23). higher wavenumber mode is that it overlaps with the first-order diamond Raman mode.

Q3. Does the Raman and XRD experiments carried out in hydrostatic environment? Different pressure environments can affect the pressure of metallization.

Measurements between samples were consistent. For BaH_2, measurement, no pressure transmitting medium was used. For Ba_8H_{46}, H_2 serves as a pressure transmitting medium. An intrinsic systematic uncertainty is assumed with stated pressures, to account for non-hydrostaticity and pressure gradients within the sample chamber.

Q4. From the insert pictures in fig3 and fig4, the authors seem to use six probes to carry out the resistance measurement. Why using this configuration?

5 and 6 probes are seen in Figures 3b and 4b, respectively. However, 2 and 4 probe measurements were carried out. More than 4 probes were created on the diamond culet in the case of electrode(s)

failing during compression, which commonly occurs. Electrode configurations were kept consistent throughout measurements. This is described further in the Supplementary Information.

Q5. The authors mentioned 'the predicted metallization pressure is 102 GPa (Tc = 1 mK) for CaH2, 177 GPa for SrH2 and 50 GPa for BaH2 (Tc = 13 mK). Can signature of superconductivity be found in BaH2 above 50GPa?

Previous works predict a superconducting T_c for BaH_2 to be less than 1 K at 50 GPa, as the reviewer pointed out (Ref. 20 - 23). In our temperature-resistance measurements, the minimum temperature achieved is 50 K. Although pressure can alter the superconducting T_c, we do not expect a T_c above this temperature for BaH_2 above 50 GPa. Therefore, we found no signature of superconductivity in BaH_2 at any pressure.

Q6. The authors used Raman spectroscopy as in-house diagnostics for both BaH_4 and Ba_8H_{46}. However, Raman spectrum of Ba_8H_{46} is featureless. The product of BaH_4 + H_2 after laser heating still remain unclear. Further analysis such as XRD should be carried out to confirm the structure.

We have previous works which structurally characterize Ba_8H_{46} (Ref. 27), so we did not consider it necessary to include XRD data in the original submission. However, this is an excellent point. We have now added a Rietveld refinement of Ba_8H_{46} at 45 GPa to the Supporting Information (Figure S4). This allows for further confirmation that the product of laser heating $BaH_4 + H_2$ to 1200 K at 45 GPa results in the synthesis of previously described Ba_8H_{46} (Ref. 27).

Q7. Resistance of the metallic BaH_2 reaches mΩ values, yet, the resistance of as claimed metallic Ba_8H_{46} plateaus around 36 Ω. What leads to this huge difference?

It is feasible that this is intrinsic to the sample. It may well be that BaH_2 is a "better" metal than Ba_8H_{46}. Comparison of the electronic properties of some common metals can reveal large differences in the resistivities. This may be due to a number of reasons, such as the electronic structure and grain boundaries within the sample.

Q8. The resistance-temperature relation presented in fig4 suggests the sample is not well connected. Does the laser-heated sample in mixed phases above 85 GPa?

Since this sample is surrounded by hydrogen, unless the sample is breaching, there is no guarantee that the connection to the electrodes will ever be excellent. There is essentially a trade-off between having a thick enough sample to be "pushed" onto the electrodes during compression whilst still maintaining enough hydrogen to form the desired phases. It can be seen in Figure 4b that an increase in pressure results in less noisy data, which supports this. There is no evidence for any mixed phase in the sample, at any pressure.

Reviewer: 2

Recommendation: This paper may be publishable, but major revision is needed; I would like to be invited to review any future revision.

Comments: The manuscript contains interesting structural and transport studies of barium dihydride under high-pressure conditions. Structural studies confirm the previously published theoretical and experimental information on pressure-induced phase transitions and changes in the stoichiometry of barium hydrides. Significantly new are only the measurements of barium hydrogen resistance and the insulator-metal transition under the influence of high pressure, also documented by optical and X measurements. The search for high-temperature superconductivity in metal hydrides has recently become widespread, and the technique of measuring electrical resistance is becoming a routine technique in many laboratories.

In my opinion, the topics and methods used in the research presented in the manuscript are not particularly innovative and the results were easy to predict in the light of existing knowledge. Nevertheless, the observation of the insulator-metal transition will probably be interesting for many researchers from the point of view of the electronic structure of metal-hydrogen systems.

We thank the referee for their insightful feedback and for believing that our work will be of interest to many researchers within the field. We will address their concerns regarding the manuscript in the following section.

I have a few comments regarding the structure of the manuscript itself.

1. The influence of hydrostatic pressure on the properties of various materials is documented by hundreds of publications, and their selection as references should strictly concern the methodology and subject matter of the presented research. Therefore, works on high-pressure properties of materials

such as silane, sulfur, or nitrogen should not be cited. There are enough works on high-pressure hydride research to place our research there.

We thank this referee for their valuable feedback and suggestions. On this point, the given references are fundamental works demonstrating insulator-metal and metal-insulator transitions which occur under high pressure. Our intent was to introduce this as a broader topic, before moving on to more specific examples which regard hydrides. We have now made this more evident and have included additional references to hydride materials which display these transitions under compression.

2. In many cases, linking high-pressure research on hydrides with their use as hydrogen or energy storage materials is irrational. This also applies to barium hydride with a hydrogen content of less than 1.5 percent by weight and high thermodynamic stability. The cited works 14-16 do not confirm the possibility of using alkaline earth hydrides as hydrogen stores. Theoretical paper 14 in Conclusions states in its only sentence that these hydrides have the potential as hydrogen storage facilities without any justification. Paper 15 only states that magnesium hydride, despite its high hydrogen content, is not suitable for storage due to the thermodynamic properties of this hydride. The same information can be found in paper 16 regarding calcium hydride. Mindless repetition of populist information addressed mainly to funders and having no scientific basis is not appropriate for authors who aspire to have their works published in serious scientific journals.

We have taken into account the reviewer's suggestions. General comments about research on hydrides as hydrogen-storing materials have been removed, along with the aforementioned references.

3. As for the References, paper 24 has no authors (sic!).

We thank the referee for pointing this out, and this has now been corrected.

4. There is no information about the origin and purity of the hydride used.

99.5% purity ABCR BaH_2 was loaded. The author guidelines for the Journal of Physical Chemistry Letters that detailed procedures can be provided in the Supporting Information, therefore, we describe this here along with the specification of the H_2 gas which was loaded.

5. Some drawings presenting the research results are difficult to read or require supplementation a) Fig. 1 smaller triangles denote previously published volumetric data that are invisible even at 5x magnification. b) Fig. 3 b, it is not clear whether the data presented concern the same sample or different measurements. Is there no data regarding pressures between 42 and 58 GPa? They would be interesting for a more accurate determination of the insulator-metal transition pressure.

Small triangles in Fig. 1 have been enlarged. The measurements presented in Fig. 3b were carried out on the same sample. This has now been clarified in the figure caption. Unfortunately, we were unable to collect temperature-resistance data between 45 and 58 GPa. Multiple cooling runs were carried out on the sample, which ultimately resulted in pressure drifting up to 58 GPa, before stabilizing, which gave us the best temperature-resistance dependency of metallic BaH_2.

6. It can be noticed that the Authors prefer to cite their works. This applies, for example, to papers 36 and 37. Optical methods for determining band gap closure have been used many times in older publications and the first ones should be cited instead. For example see "High-pressure study of ScH3: Raman, infrared, and visible absorption spectroscopy, Tetsuji Kume, Hiroyuki Ohura, Tomoo Takeichi, Ayako Ohmura, Akihiko Machida, Tetsu Watanuki, Katsutoshi Aoki, Shigeo Sasaki, Hiroyasu Shimizu, and Kenichi Takemura, Phys. Rev. B 84, 064132, 2011, and references given there.

Following the reviewer's suggestion, we have removed newer references which may be deemed inappropriate. However, the procedure used to calculate the band gap from absorbance data was carefully followed the procedure used by Goncharov *et al.* in Ref. 33 (former Ref. 36). Along with this, Ref. 33 is significantly older than there references suggested by the reviewer, therefore, we have kept this as a reference and not included the suggested references.

7. In several places the authors use the term insulator-metallic transition instead of insulator-metal

Metallic has been changed to *metal*, where relevant, as suggested.

8. In the sentence on Page 10, lines 48,49 "by" is probably missing.

We thank the reviewer for pointing this out. We believe there was a "to" missing, which has now been added to the aforementioned sentence.

Taking into account the above comments regarding the innovativeness of the work and its presentation, I cannot recommend the manuscript for publication in the Journal of Physical Chemistry Letters in the present form. I suggest that, after taking into account my comments, the manuscript should be submitted to the Journal of Phys, Chem. C were it will be more suitable regarding the scientific level.

The reviewer states that the insulator-metal transition is interesting regarding metal-hydrogen systems. We are in absolute agreement with this, and this is the main point of the paper. Such research has utmost relevance to current topics within the high-pressure field, considering the current excitement about hydrides within the community. Therefore, we respectfully disagree with the reviewer and will resubmit the manuscript to the Journal of Physical Chemistry Letters.

Stylistic changes

1. Graphics: One or more of your figures and tables includes a reference citation. Please confirm that this pertains only to data and not the figure itself. If it pertains to the use of a published image, permissions must be secured for any graphics NOT originally published by ACS or for Open Access content which permits reuse with credit only. Permission is needed if you are using another publisher's or copyright owner's figures/tables verbatim, adapting/modifying them, or using them in part. If the images are from an Open Access publisher that does not require permission for reuse, please confirm.

We can confirm that the relevant citations pertain only to data included in the figures, and not the figures themselves.

2. Author Affiliations: Please include postal codes/country in the author affiliations in the publication file(s).

Postal codes and countries for each affiliation have been added.

3. Supporting Information: Please number SI pages in the following format: "S1, S2..."

SI pages are now labelled accordingly.

jz-2023-02704c.R2

Name: Peer Review Information for "Pressure-Induced Metallization of BaH₂ and the Effect of Hydrogenation"

Second Round of Reviewer Comments

Reviewer: 1

Comments to the Author

The authors have addressed all of my concerns, I recommend the manuscript to be accepted.

Reviewer: 2

Comments to the Author

I am pleased that some of my critical comments were accepted by the authors.

The lack of data on the temperature dependence of the hydride's electrical resistance in the pressure range of 45-58 GPa indicates that only one cycle of measurements was performed. In the case of such a dramatic change in trend, failure to check the repeatability of this phenomenon is a serious omission that reduces the scientific value of the entire work. The claim “Electrical transport measurements as a function of pressure and temperature confirm the insulator-metal transition in BaH2 at 57(?) GPa” is baseless.

The authors' reaction to my comment about the energy gap in the materials requires further comment from me. If the authors care about the historical aspect of measurements of this quantity, the most appropriate references are the works of A. S. Balchan; H. G. Drickamer J. Chem. Phys. 34, 1948–1949 (1961) or M. Pasternak, J. N. Farrell, and R. D. Taylor Phys, Rev, Lett, 58 (5), 1987.

Goncharov's work does not concern metallization but the insulator-to-semiconductor transition, and there is no extrapolation of the size of the gap allowing the assessment of the metallization pressure. Again, it is difficult not to notice an attempt to quote the work of a colleague and former collaborator of one of the authors, which has little to do with the examined object.

I understand the authors' desire to publish in Physical Review Letters, but I still suggest sending the work to Phys. Rev.C.

Author's Response to Peer Review Comments:

Reply to the Referees on "Pressure-Induced Metallization of BaH_2 and the Effect of Hydrogenation", H. A. Shuttleworth *et al.*

November 6, 2023

We would like to thank the Reviewer 1 for recommending our manuscript for publication.

Reviewer 2:

I am pleased that some of my critical comments were accepted by the authors. The lack of data on the temperature dependence of the hydride's electrical resistance in the pressure range of 45-58 GPa indicates that only one cycle of measurements was performed. In the case of such a dramatic change in trend, failure to check the repeatability of this phenomenon is a serious omission that reduces the scientific value of the entire work. The claim "Electrical transport measurements as a function of pressure and temperature confirm the insulator-metal transition in BaH2 at 57(?) GPa" is baseless.

We believe that the reviewer has shifted the goalpost, and is now contradicting what was previously said. In the first round of comments, the reviewer stated:

Significantly new are only the measurements of barium hydrogen resistance and the insulator-metal transition under the influence of high pressure, also documented by optical and X measurements. The search for high-temperature superconductivity in metal hydrides has recently become widespread, and the technique of measuring electrical resistance is becoming a routine technique in many laboratories. .... Nevertheless, the observation of the insulator-metal transition will probably be interesting for many researchers from the point of view of the electronic structure of metal-hydrogen systems.

In the previous round of comments, the reviewer stated that the experiments are routine and commented on the fact that we have conducted more than one independent experiment, using very different techniques, all of them leading to the same conclusion: barium hydride becomes metallic above 57 GPa. We have conducted (1) reflectivity and (2) absorption measurements - both are consistent with the fact that BaH_2 becomes metallic at above 57 GPa; (3) the resistance of the sample drops to the typical metallic values and plateaus at pressures above 57 GPa, (4) the temperature-resistance measurements show typical semiconducting behaviour below 57 GPa and typical metallic behaviour above. We do not believe that the conclusion of four independent techniques which support our claim could lead to a baseless claim, and it appears that in the first round of comments, the reviewer recognised this too.

The authors' reaction to my comment about the energy gap in the materials requires further comment from me. If the authors care about the historical aspect of measurements of this quantity, the most appropriate references are the works of A. S. Balchan; H. G. Drickamer J. Chem. Phys. 34, 1948–1949 (1961) or M. Pasternak, J. N. Farrell, and R. D. Taylor Phys, Rev, Lett, 58 (5), 1987. Goncharov's work does not concern metallization but the insulator-to-semiconductor transition, and there is no extrapolation of the size of the gap allowing the assessment of the metallization pressure. Again, it is difficult not to notice an attempt to quote the work of a colleague and former collaborator of one of the authors, which has little to do with the examined object.

We thank the reviewer for their further suggestions for additional references, and we have now included these key articles in the manuscript. However, we would still like to reiterate that the work by Goncharov *et al.* (Ref 33) describes in detail the method which we have followed and used to estimate the band gap, and we do not agree that it is simply "*an attempt to quote the work of a colleague*". To state "*which has little to do with the examined object*" is completely baseless, and raises the question of whether the reviewer has read our paper carefully.

I understand the authors' desire to publish in Physical Review Letters, but I still suggest sending the work to Phys. Rev.C.

We would like to remind the reviewer that the journal they are reviewing for is JPCL, the journal to which we have submitted our manuscript, not PRL. Although we appreciate the feedback given by reviewer 2, the recommendation not to publish in PRL is irrelevant.

Stylistic changes

1. Graphics: One or more of your figures and tables includes a reference citation. Please confirm that this pertains only to data and not the figure itself. If it pertains to the use of a published image, permissions must be secured for any graphics NOT originally published by ACS or for Open Access content which permits reuse with credit only. Permission is needed if you are using another publisher's or copyright owner's figures/tables verbatim, adapting/modifying them, or using them in part. If the images are from an Open Access publisher that does not require permission for reuse, please confirm.

We can confirm that the relevant citations pertain only to data included in the figures, and not the figures themselves.
